# Mucosal gene expression changes induced by anti‐TNF treatment in inflammatory bowel disease patients

**DOI:** 10.1002/ddr.21566

**Published:** 2019-07-19

**Authors:** Elena Milanesi, Maria Dobre, Teodora E. Manuc, Gabriel Becheanu, Cristian G. Tieranu, Elena M. Ionescu, Mircea Manuc

**Affiliations:** ^1^ National Institute of Pathology “Victor Babeş” Bucharest Romania; ^2^ “Fundeni” Clinical Institute Bucharest Romania; ^3^ “Elias” Emergency University Hospital Bucharest Romania; ^4^ “Carol Davila” University of Medicine and Pharmacy Bucharest Romania

**Keywords:** 5‐ASA, anti‐TNF, Crohn disease, ulcerative colitis

## Abstract

In the last two decades anti‐tumor necrosis factor (anti‐TNF) therapy for inflammatory bowel disease (IBD) has been widely used to induce and maintain clinical and endoscopical remission, completely changing management of the disease. In this study, we aimed to identify gene expression changes in inflamed mucosa from Crohn's disease and ulcerative colitis patients treated with 5‐aminosalicylic acid (5‐ASA) (*N* = 25) or anti‐TNF agents (*N* = 12) compared to drug‐free IBD patients (*N* = 12) and non‐IBD control subjects (*N* = 18). The mucosal expression of 84 genes previously associated with IBD was evaluated by qPCR. We found that both therapeutic regimens induce a decrease in LCN2, NOS2, and TFF1, the levels of which are overexpressed in drug‐free patients compared to non‐IBD control subjects. Interestingly, a stronger effect of anti‐TNF drugs was observed on LCN2 and TFF1 levels. However, 5‐ASA seems to induce a more robust reduction of NOS2 expression. Moreover, we found that anti‐TNF treatment significantly increased ABCB1, leading to levels similar to those found in non‐IBD control subjects.

## 1 INTRODUCTION

Inflammatory bowel diseases (IBDs) are chronic inflammatory conditions comprising Crohn's disease (CD) and ulcerative colitis (UC). The relapsing and remitting course of these diseases negatively affects quality of life because disease flares occur in a random way and are mostly unpredictable (Liverani, Scaioli, Digby, Bellanova, & Belluzzi, 2016). Classical therapies include corticosteroids, thiopurines, and aminosalicylates (5‐ASA), which have been used to treat IBD for decades. Among these, 5‐ASA has an excellent safety profile (Loftus, Kane, & Bjorkman, 2004) with few minor side effects and can be very effective for treating mild to moderate UC, but not for treating CD (Williams, Panaccione, Ghosh, & Rioux, 2011). Limited use of corticosteroids due to severe adverse effects and biological advancements has created the premises for anti‐TNF therapy. In the last two decades, apart from anti‐tumor necrosis factor (anti‐TNF) agents, other new therapeutic molecules targeting several immune pathways have also been developed. The use of these drugs has improved long‐term outcomes for both UC and CD (Reinisch et al., 2012; Schnitzler et al., 2009) with a significant increase in the economic burden on many national health care systems (Reinglas, Gonczi, Kurt, Bessissow, & Lakatos, 2018). If initially the therapeutic aim was to limit patient symptoms, current treatment goals for IBD include steroid‐free remission, endoscopic healing, and lower surgery rates (Atreya & Neurath, 2017). However, approximately 30% of patients do not respond to treatment, and among those who do, the loss of response at 12 months of therapy occurs in 23–46% of cases (Ben‐Horin & Chowers, 2011). Consequently, identifying predictors of nonresponse and selecting treatment according to molecular perturbations can improve overall IBD disease management.

Mucosal gene expression in IBD is of particular interest, since a disruption in the epithelial intestinal barrier is at the epicenter of disease onset. Molecular analysis of biomarkers in intestinal biopsies is feasible and provides a reproducible method for measuring inflammation specific to localization, since it is highly sensitive and dynamic. Through this method, we can observe not only pathophysiological processes involved in the chronic inflammatory process, but also the effect of treatment, another issue in IBD disease management.

Romania is a country with low IBD incidence and prevalence, where IBD diseases are usually milder, fewer cases are severe enough to require surgical interventions, a lower number of complicated diseases are experienced, and patients respond well to classical treatment (Gheorghe et al., 2004). However, in the past few years a rise in the number of IBD cases in Eastern Europe has been noted (Cristina, Anda Carmen, & Luana, 2017). Few studies researching molecular mucosal profiling have been performed in this area (Dobre et al., 2018; Velikova et al., 2017).

The aim of our study was to identify gene expression changes in inflamed mucosa from IBD patients undergoing 5‐ASA or anti‐TNF treatment compared to those in drug‐free IBD patients and non‐IBD control subjects.

## 2 METHODS

### 2.1 Patients

Biopsies of endoscopically inflamed and noninflamed colonic mucosa were obtained from 49 IBD patients and 18 non‐IBD control subjects, respectively. Patients and controls were recruited at the Fundeni Clinical Institute and at the Department of Gastroenterology and Hepatology of “ELIAS” Emergency University Hospital in Bucharest, Romania. Written informed consent was obtained from all participants prior to biopsy sampling. The study was approved by the local ethics committees. The diagnosis of UC and CD had been made according to guidelines from the European Crohn's and Colitis Organization (ECCO) (Magro et al., 2017). Exclusion criteria for non‐IBD control subjects are previously described (Dobre et al., 2018). At the time of recruitment, 12 patients (24.5%) were drug‐free, 25 (51%) were in treatment with 5‐aminosalicylic acid (5‐ASA), and 12 (24.5%) were receiving anti‐TNF treatment (anti‐TNF). Among the treated patients, 5 presented endoscopically as in remission, and 10 were in the active phase. All patients under treatment had had a clinical response according to a MAYO partial score and CDAI score. The characteristics of the patients and non‐IBD control subjects are reported in Table 1.

**Table 1:** Characteristics of the individuals involved in the study

IBD patients	*N* = 49
*Age* (mean ± *SD*)	44.48 ± 14.34
*Sex* (%M)	69%
*Treatment and duration*	
Drug free	24.50%
5‐ASA	51% (12–144 months)
Anti‐TNF	24.5% (3–48 months)
*Activity of the disease*	
Remission	26.5%
Active	73.5%

Non‐IBD controls	*N* = 18
*Age* (mean ± *SD*)	47.11 ± 17.47
*Sex* (%M)	44%

*Note*. Socio demographic and clinical characteristics of individuals involved in the study.

### 2.2 Total RNA isolation and qPCR

Total RNA isolation from fresh‐frozen tissues was performed using RNeasy mini kit (Qiagen, Germany), and RNA quality and quantity were assessed by spectrophotometric method (NanoDrop 2000, Thermo Scientific). Reverse transcription using 600 ng of RNA was performed with the RT2 First Strand Kit (Qiagen). The expression of the 84 key genes was evaluated with Human Crohn's Disease RT2 Profiler PCR Array (PAHS‐169Z, Qiagen), as previously reported (Ţieranu et al., 2017), using SYBR Green chemistry on the ABI‐7500 fast instrument (Applied Biosystems). The Ct value of each gene was normalized according to the delta Ct method on the geometric mean of two housekeeping genes, GAPDH and HPRT1, as previously reported (Dobre et al., 2018).

### 2.3 Statistical analysis

Categorical variables were tested by means of the chi‐square test and continuous variables with the *t* test. Normality of data distribution of each gene level was evaluated using the Shapiro–Wilk test. Since data were not normally distributed, differences in gene expression among the groups were evaluated using the nonparametric Kruskal–Wallis test. Statistical analysis was performed using the Statistical Package for Social Science (SPSS version 17.0).

## 3 RESULTS

No difference in age (*p* = .59) and sex distribution (*χ* ^2^ = 3.502, *p* = .061) was observed between IBD patients and non‐IBD control subjects. When considering patients, a difference in the treatment duration was found between 5‐ASA (45.16 months ± 34.65) and anti‐TNF (20.08 months ± 18.90) groups (*p* = .025).

**Figure 1:**
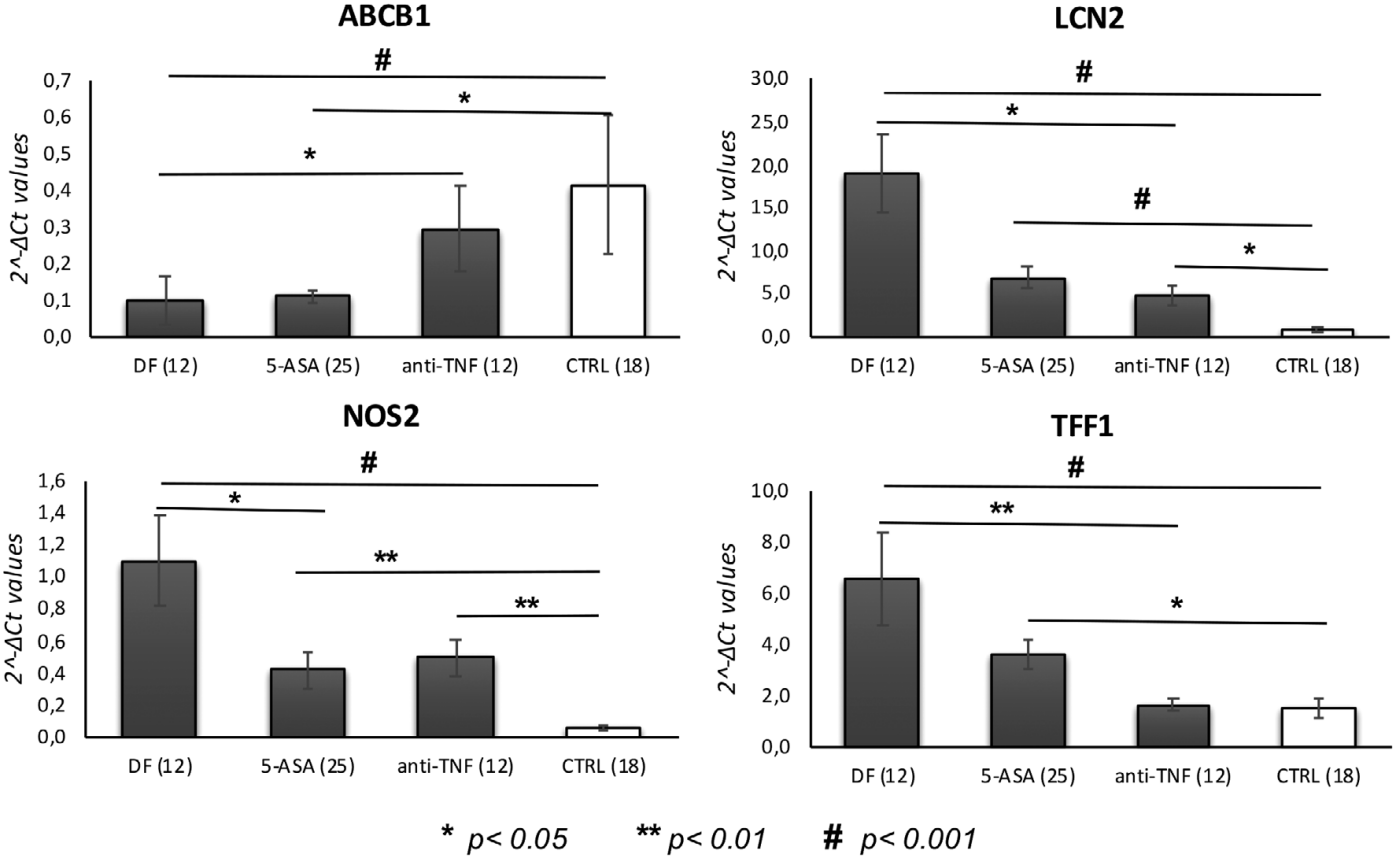
Bar graphs represent the mean of the 2^−Δ*Ct*^ values, and error bars represent the standard error. The graphs show the genes differentially expressed among the groups (DF = drug‐free IBD patients; 5‐ASA = IBD patients treated with 5‐aminosalicylic acid; anti‐TNF = IBD patients treated with anti‐TNF drugs; CTRL = non‐IBD control subjects)

Analysis of the differences in mRNA levels in IBD patients with different therapeutic regimens, drug‐free IBD patients, and non‐IBD control subjects revealed that the expression of five genes was modulated by treatment. Results of the comparisons among the groups are reported in Table 2 and Figure 1.

**Table 2:** Gene expression results

Gene	2^−Δ*Ct*^ values				*p* value	
	Drug free	5‐ASA	Anti‐TNF	Controls		
*ABCB1*	0.09 ± 0.06	0.11 ± 0.02	0.29 ± 0.11	0.42 ± 0.19	DF vs. anti‐TNF DF vs. CTRL 5‐ASA vs. CTRL	0.023 <0.001 0.041
*LCN2*	18.96 ± 4.48	6.84 ± 1.31	4.73 ± 1.22	0.71 ± 0.23	DF vs. anti‐TNF DF vs. CTRL 5‐ASA vs. CTRL Anti‐TNF vs. CTRL	0.035 <0.001 <0.001 0.037
*NOS2*	1.10 ± 0.28	0.42 ± 0.12	0.49 ± 0.12	0.06 ± 0.02	DF vs. CTRL DF vs. 5‐ASA 5‐ASA vs. CTRL Anti‐TNF vs. CTRL	<0.001 0.034 0.007 0.003
*TFF1*	6.54 ± 1.77	3.57 ± 0.56	1.63 ± 0.25	1.49 ± 0.38	DF vs. CTRL DF vs. anti‐TNF 5‐ASA vs. CTRL	<0.001 0.009 0.016

*Note*. Levels of the transcripts (as 2^−Δ*Ct*^ values) differentially expressed among the groups. Genes are arranged in alphabetic order. CTRL = controls; DF = drug free.

Both treatments, 5‐ASA and anti‐TNF, increased the levels of ABCB1 that were significantly reduced in drug‐free patients compared with non‐IBD controls. A stronger effect on the expression of this gene was observed in patients taking anti‐TNF agents. In addition, these drugs reduced LCN2, NOS2, and TFF1 expression levels. Of note, anti‐TNF treatment was found to significantly modulate the levels of ABCB1 and TFF1, leading to levels similar to those of non‐IBD controls. No significant correlations were found between the duration of treatment and the levels of the significant genes.

## 4 DISCUSSION

In this study, we used a target gene expression approach to examine genes implicated in inflammation, apoptosis, immune response, cellular adhesion, and tissue remodeling in order to assess changes in the colonic mucosa from IBD patients under different therapeutic regimens. While transcriptional studies of intestinal specimens comparing IBD patients and controls have been extensively used to identify biomarkers, studies comparing the molecular signature of patients under different treatments are underrepresented.

Our findings showed that both 5‐ASA and anti‐TNF agents are able to decrease the mucosal expression of LCN2, NOS2, and TFF1, the levels of which are overexpressed in untreated patients compared to non‐IBD controls. Interestingly, a stronger effect of anti‐TNF drugs was observed on LCN2 and TFF1, where treatment restored the levels to those observed in the nonaffected individuals. Further, 5‐ASA seems to have a better effect on NOS2 expression, even though differences with non‐IBD controls remain significant. Moreover, we found that anti‐TNF treatment significantly increased the expression of ABCB1, leading to levels similar to those found in non‐IBD control subjects.

LCN2 and NOS2 genes codify for two antimicrobial peptides (AMPs) named neutrophil gelatinase‐associated lipocalin (NGAL) and nitric oxide synthase 2, respectively. Antimicrobial peptides act on the innate immunity, protecting intestinal mucosa against microorganisms, thus maintaining its barrier integrity.

LCN2/NGAL limits bacterial growth, sequestering iron‐containing siderophores, whereas NOS2 produces nitric oxide, a reactive free radical, which acts as a biologic mediator in antimicrobial, neurotransmission, and anti‐tumoral activities.

Different studies have indicated that both LCN2/NGAL and NOS2 are strongly upregulated in the mucosa of UC and CD patients (Arijs et al., 2009; Dobre et al., 2017; Lawrance, Fiocchi, & Chakravarti, 2001; Nielsen et al., 1996; Thorsvik et al., 2018). Of note, NGAL protein has been suggested as a promising biomarker for IBD, since its fecal levels, if elevated, are significantly associated with disease activity and severity (Thorsvik et al., 2017; Yeşil et al., 2013; Zinkevich et al., 2014). Increased levels of LCN2/NGAL were observed in the serum of active UC patients (Stallhofer et al., 2015), and treatment with infliximab resulted in a decrease of these levels (de Bruyn et al., 2014). Moreover, high LCN protein concentration was identified in the urine of CD patients, which was significantly reduced after a single high dose of infliximab (Bolignano et al., 2010). This interesting finding can also be observed in our study, where LCN2 expression was lower in the treated group.

NOS2 is an enzyme inducible by a combination of lipopolysaccharides and specific inflammatory cytokines and is responsible for the production of nitric oxide (NO). Nitric oxide regulates intestinal epithelial cells and protects barrier integrity, supporting tight junctions. Moreover, it triggers a redox imbalance through a free radical mechanism that initiates lipid and protein oxidation reactions (Mu, Yu, & Kitts, 2019). Nitric oxide synthase activity has been shown to be increased in the colon mucosa from both UC and CD patients (Guihot et al., 2000).

Genetic variants within the NOS2 gene have been associated with IBD susceptibility in different populations (Martín et al., 2007; Senhaji et al., 2017), and increased mRNA levels were found in the colonic mucosa of patients with active UC, compared to patients with inactive UC and controls, and in CD patients compared to controls (Coburn et al., 2016). Recently, Luther and collaborators found that colonic expression of NOS2 was elevated in patients with loss of response to TNFα‐antagonist compared to responders (Luther et al., 2018).

Another gene found upregulated in untreated patients and modulated by anti‐TNF and 5‐ASA treatments is TFF1. It belongs to the trefoil family, which comprises TFF1, TFF2, and TFF3. They are stable secretory proteins widely expressed in the gastrointestinal mucosa, and their role in the protection and repair of epithelial surfaces has been confirmed (Kjellev, 2009). In response to acute mucosal injury, TFFs accelerate cell migration to seal the damaged area from luminal contents, whereas chronic inflammation leads to increased TFF expression to prevent further progression of the disease (Aihara, Engevik, & Montrose, 2017). An immunohistochemistry study on colorectal biopsy specimens found that TFF1 was expressed in severe UC patients, whereas no expression was detected in normal tissue (Longman et al., 2006). The colonic expression of this trefoil protein was also observed in children with IBD (Shaoul, Okada, Cutz, & Marcon, 2004). The expression of the trefoil factors has also been measured in serum, finding increased concentration of TFF1 and TFF3 in IBD patients (Vestergaard et al., 2004). Moreover, TFF3 concentration in serum correlated with clinical and biochemical parameters of disease activity in UC patients (Grønbaek, Vestergaard, Hey, Nielsen, & Nexø, 2006).

In our cohort ABCB1 was the single transcript found downregulated in drug‐free patients and increased by anti‐TNF treatment.

ABCB1, also known as the multidrug resistance 1 (MDR1) gene, codifies for P‐Glycoprotein 1, which is an ATP‐dependent transmembrane pump with the role of moving drugs from the intracellular to the extracellular domain. ABCB1 is well expressed in the normal gastrointestinal tract, and its loss of function, as well as decreased expression levels in the gut, have been indicated to contribute to the pathogenesis of IBD (Ho et al., 2018). Furthermore, although several genetic studies have been conducted to evaluate the association between IBD susceptibility and three SNPs considered to be the most clinically relevant (G2677T/A, C3435T, and C1236T), results are conflicting (Petryszyn & Wiela‐Hojeńska, 2018).

Few studies have evaluated the involvement of ABCB1 in anti‐TNF agent response. No correlation between ABCB1 gene variants and response to infliximab was observed in Italian IBD patients (Palmieri et al., 2005) or in Hungarian patients (Fischer et al., 2007). In our study, we found that the low levels of ABCB1 in patients are significantly restored by anti‐TNF treatment, reaching, in treated patients, levels similar to those of non‐IBD controls. No study has evaluated the putative modulation of ABCB1 in mucosa of IBD patients by anti‐TNF treatment, and a single in vitro study on Caco‐2 cell lines showed that in vitro TNFα treatment induced diminution of MDR1 mRNA levels (Belliard, Lacour, Farinotti, & Leroy, 2004).

A potential limitation of our study is its cross‐sectional character. Moreover, the size of our population was relatively small, which precluded us from further subgroup analysis (UC and CD separately).

In conclusion, taking into account the possible limitations of this study, we found a pattern of genes, the ectopic expression of which in IBD mucosa seems to be better modulated by anti‐TNF than by 5‐ASA drugs.

## CONFLICT OF INTEREST

The authors declare that they have no conflict of interests.
